# Genomic value prediction for quantitative traits under the epistatic model

**DOI:** 10.1186/1471-2156-12-15

**Published:** 2011-01-26

**Authors:** Zhiqiu Hu, Yongguang Li, Xiaohui Song, Yingpeng Han, Xiaodong Cai, Shizhong Xu, Wenbin Li

**Affiliations:** 1 Department of Botany and Plant Science, University of California, Riverside, California, 92521, USA; 2 Soybean Research Institute (Chinese Education Ministry's Key Laboratory of Soybean Biology), Northeast Agricultural University, 150030 Harbin, PR China; 3 Dept. of Electrical & Computer Engineering, University of Miami 1251 Memorial Drive, Coral Gables, FL 33146, USA

## Abstract

**Background:**

Most quantitative traits are controlled by multiple quantitative trait loci (QTL). The contribution of each locus may be negligible but the collective contribution of all loci is usually significant. Genome selection that uses markers of the entire genome to predict the genomic values of individual plants or animals can be more efficient than selection on phenotypic values and pedigree information alone for genetic improvement. When a quantitative trait is contributed by epistatic effects, using all markers (main effects) and marker pairs (epistatic effects) to predict the genomic values of plants can achieve the maximum efficiency for genetic improvement.

**Results:**

In this study, we created 126 recombinant inbred lines of soybean and genotyped 80 makers across the genome. We applied the genome selection technique to predict the genomic value of somatic embryo number (a quantitative trait) for each line. Cross validation analysis showed that the squared correlation coefficient between the observed and predicted embryo numbers was 0.33 when only main (additive) effects were used for prediction. When the interaction (epistatic) effects were also included in the model, the squared correlation coefficient reached 0.78.

**Conclusions:**

This study provided an excellent example for the application of genome selection to plant breeding.

## Background

Genome selection refers to a method for genomic value prediction using markers of the entire genome [1,2]. It is effective for genetic improvement of quantitative traits that are controlled by multiple quantitative trait loci (QTL). Some traits may be controlled by only a few QTL and marker assisted selection using only the few detected QTL can be effective. However, most quantitative traits are determined by multiple QTL and their interactions. Marker assisted selection using only a few detected loci may not be efficient for these traits. Using all QTL collectively to predict the breeding values of individual plants can outperform the traditional marker assisted selection [3,4]. However, there might be some trade off between the numbers of QTL included in the model and the efficiency of prediction. Cross validation can help us determine the optimal number of QTL included in the model to maximize the efficiency of genome selection.

The importance of epistasis in genetic determination may vary across different species. In agricultural crops, most quantitative traits in barley do not have a strong basis of epistatic effects [5]. However, epistasis has been shown to be important in QTL studies in rice [6-8]. Dudley and Johnson [9] found that epistatic effects are more important than additive effects in determination of oil, protein and starch contents of corn. They concluded that epistasis is an important contributor to the long term response to selection of these quantitative traits.

The number of somatic embryos is an important trait for consideration in soybean breeding program because it is directly related to the plant regeneration system that is essential for effective gene transfer. The capacity of plant regeneration through immature embryo culture of soybean is genetically determined, reflected by significant variation across different lines (from 0% to 100% of regeneration). The genetic knowledge of the regeneration trait based on immature embryo culture and the discovery of molecular markers associated with regeneration will offer a great opportunity to develop efficient elite inbred lines with increased regeneration capacity. However, studies on the genetic basis of embryogenesis are lacking. There is no information available about the role of epistasis. In this study, we used advanced statistical methods to investigate not only the main effects but also pair-wise interaction (epistatic) effects for soybean somatic embryogenesis.

Statistical methods for QTL mapping are available but mainly for individual marker (main effect) analysis and individual marker pair (epistatic effect) analysis [10-12]. The epistatic model analysis in corn conducted by Dudley and Johnson [9] is an example of such studies. Recently, Xu and Jia [5] applied a Bayesian shrinkage method, called the empirical Bayesian method by Xu [13], to evaluate all markers and marker pairs of the whole genome to estimate the genomewide epistatic effects. The empirical Bayesian method [13] provides better estimation of the epistatic effects because all effects are estimated simultaneously in a single model. This method has not been applied to QTL study in other species. The method can evaluate many effects simultaneously rather than separately. When the number of model effects is larger than the sample size, the model can fit the data perfectly, but may loose the predictive value. Cross validation is an effective approach for model checking and variable selection [14] and has been used for genome prediction in plants [15] and animals [16]. This study provides another example of successful use of cross validation for genome selection.

## Result

### Main effect model

**Figure 1:**
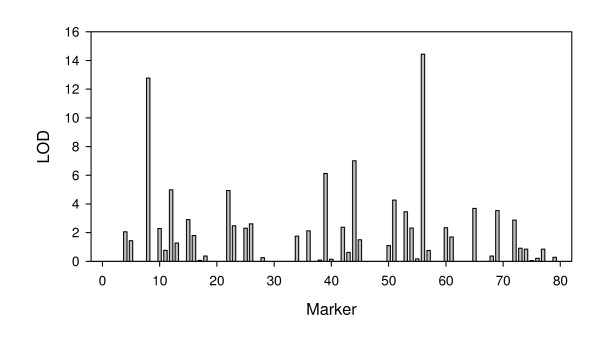
**LOD scores for the 80 markers (main effects) obtained from the empirical Bayesian analysis**.

The numerical codes (marker IDs) and names of the 80 markers are given in Table 1 along with the positions and the linkage groups. For example, marker 74 (M74) in the model has a marker name Satt579, which is located in position 149.39 cM of linkage group 1. The numerical codes allow an easy way to make a graphical presentation of the results. The LOD (log of odds) scores of all the 80 markers (main effects) are plotted in Figure 1. Four markers have LOD scores greater than 6, which are M56, M8, M44 and M39. The marker with the largest LOD score (M56) explained 13% of the phenotypic variance. The marker with the smallest LOD score (M39) of the four explained about 5% of the phenotypic variance. The four markers collectively explained about 30% of the phenotypic variance. We used the leave-one-out cross validation analysis [17] to select the top 27 markers (see next paragraph for the result of cross validation) and found that the 27 markers collectively explained about 33% of the phenotypic variance. The marker effects and their LOD scores are presented in Table 2.

**Table 1:** Names, positions (cM) and linkage groups (LG) of the 80 markers (M1-M80) presented in Tables 2 and 3, Figures 1, 2, 3 and 5.

**Marker ID**	**Marker name**	**cM**	**LG**	**Marker ID**	**Marker name**	**cM**	**LG**
	
M1	Satt005	0.00	1	M11	Satt123	0.00	10
M74	Satt579	149.39	1	M73	Satt576	70.86	10
M37	Satt290	241.33	1	M9	Satt094	138.02	10
M70	Satt537	586.72	1	M6	Satt052	0.00	11
M4	Satt032	0.00	2	M44	Satt353	89.85	11
M58	Satt436	196.10	2	M61	Satt469	169.72	11
M79	Satt605	272.50	2	M23	Satt181	169.72	11
M69	Satt532	388.23	2	M57	Satt434	293.93	11
M75	Satt584	0.00	3	M41	Satt317	465.41	11
M31	Satt234	152.25	3	M72	Satt568	670.93	11
M49	Satt387	244.95	3	M15	Satt150	0.00	12
M3	Satt022	304.72	3	M63	Satt494	345.39	12
M32	Satt247	0.00	4	M26	Satt201	455.66	12
M43	Satt337	345.39	4	M22	Satt175	538.75	12
M13	Satt137	439.85	4	M71	Satt567	588.30	12
M62	Satt475	545.69	4	M56	Satt427	0.00	13
M47	Satt375	545.69	4	M12	Satt130	91.38	13
M33	Satt264	609.52	4	M25	Satt199	190.84	13
M5	Satt046	670.69	4	M65	Satt505	259.01	13
M34	Satt268	0.00	5	M76	Satt594	604.40	13
M28	Satt213	345.39	5	M24	Satt195	0.00	14
M78	Satt602	434.04	5	M19	Satt161	68.96	14
M52	Satt411	528.24	5	M38	Satt294	170.89	14
M48	Satt384	600.86	5	M51	Satt399	255.15	14
M30	Satt231	674.74	5	M68	Satt529	0.00	15
M77	Satt598	743.69	5	M29	Satt215	72.32	15
M40	Satt307	0.00	6	M67	Satt528	417.71	15
M36	Satt286	41.97	6	M17	Satt158	0.00	16
M53	Satt422	88.51	6	M27	Satt206	345.39	16
M35	Satt281	164.63	6	M14	Satt146	0.00	17
M66	Satt520	252.18	6	M18	Satt160	89.85	17
M80	GMA	300.27	6	M54	Satt425	197.04	17
M7	Satt082	0.00	7	M46	Satt374	273.57	17
M50	Satt397	345.39	7	M16	Satt155	0.00	18
M21	Satt168	0.00	8	M39	Satt300	76.49	18
M60	Satt467	67.12	8	M42	Satt330	0.00	19
M10	Satt122	247.35	8	M59	Satt451	98.09	19
M45	Satt373	0.00	9	M2	Satt008	N/A	N/A
M64	Satt495	345.39	9	M8	Satt085	N/A	N/A
M20	Satt166	462.25	9	M55	Satt426	N/A	N/A

**Table 2:** The empirical Bayesian estimates of the top 27 marker (main) effects.

Marker	Variance	Effect	StdErr	LOD	p- value	H
M56	1.0181	1.0016	0.1228	14.44	0.0000	0.1303
M8	0.7492	0.8581	0.1118	12.77	0.0000	0.0980
M44	0.4744	-0.6783	0.1193	7.01	0.0000	0.0562
M39	0.4328	-0.6466	0.1217	6.13	0.0000	0.0525
M12	0.3084	-0.5437	0.1134	4.98	0.0000	0.0384
M22	0.3108	0.5455	0.1143	4.94	0.0000	0.0399
M51	0.3114	0.5444	0.1228	4.26	0.0000	0.0375
M65	0.2437	0.4797	0.1163	3.69	0.0000	0.0304
M69	0.2126	-0.4477	0.1109	3.53	0.0001	0.0271
M53	0.2095	0.4439	0.1111	3.46	0.0001	0.0241
M15	0.2217	-0.4543	0.1240	2.91	0.0003	0.0270
M72	0.1711	-0.3989	0.1095	2.88	0.0003	0.0210
M26	0.1781	-0.4054	0.1167	2.62	0.0005	0.0203
M23	0.1909	-0.4192	0.1241	2.47	0.0007	0.0222
M42	0.1915	0.4188	0.1267	2.37	0.0010	0.0232
M60	0.1662	-0.3900	0.1185	2.35	0.0010	0.0191
M54	0.1506	-0.3712	0.1133	2.33	0.0011	0.0173
M25	0.1437	0.3625	0.1111	2.31	0.0011	0.0181
M10	0.1336	0.3492	0.1075	2.29	0.0012	0.0166
M36	0.1433	-0.3605	0.1150	2.13	0.0017	0.0167
M4	0.1363	0.3513	0.1138	2.07	0.0020	0.0158
M16	0.1171	-0.3233	0.1121	1.80	0.0039	0.0135
M34	0.1141	0.3187	0.1120	1.76	0.0044	0.0133
M61	0.0982	-0.2951	0.1055	1.70	0.0052	0.0118
M45	0.1027	-0.2997	0.1136	1.51	0.0083	0.0116
M5	0.0961	-0.2890	0.1121	1.44	0.0099	0.0106
M13	0.0802	-0.2619	0.1081	1.27	0.0154	0.0093

The last column (H) gives the proportion of phenotypic variance contributed by each marker. The markers are sorted based on their LOD scores.

**Figure 2:**
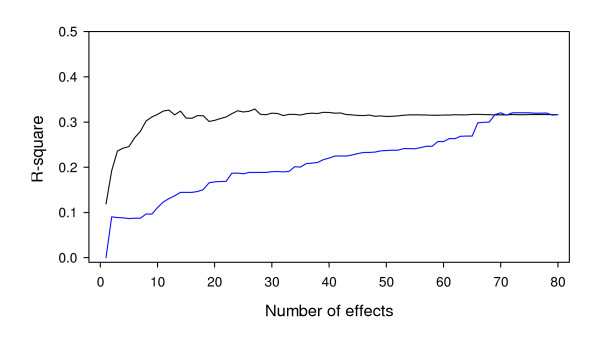
**The r-square between the phenotypic values of somatic embryogenesis of soybean and their predictions by the leave-one-out cross validation analysis**. The black curve represents the change of squared correlation coefficient by increasing the number of sorted markers (from the strongest to the weakest) in the model. The blue curve shows the change of squared correlation coefficient by increasing the number of randomly selected markers in the model. The squared correlation coefficient reaches its maximum at 0.33 when 27 markers with the largest LOD scores are included in the model.

We now examine the result of cross validation. All the 80 markers were ordered from the largest to the smallest according to the LOD scores. We then used the cross validation analysis to calculate the squared correlation coefficient between the observed phenotypic values and the predicted genomic values. For the prediction, we examined the change of the r-square with the number of markers included in the model for prediction. The result is plotted in Figure 2 (the curve in black). When only the marker with the largest LOD was included, the r-square was only 0.12. As the number of markers increased, the r-square started to increase until it reached 0.32 when 15 markers were included. The r-square then dropped and raised again to 0.33 when the top 27 markers (based on LOD scores) were included. Further increase of the number of markers caused a progressive decrease of the r-square until it reached 0.3 when all the 80 markers were included. This explains why we presented the top 27 markers in the previous paragraph. The cross validation helped us determine the optimal number of markers for inclusion. Once the optimal number of markers is reached, further inclusion of markers with small effects actually introduced noise and thus decreased the r-square value. To make sure that the plot was not generated due to any artifacts, we also included markers randomly rather than selectively based on their LOD scores. The corresponding profile of the r-square is also shown in Figure 2 (the curve in blue). We can see that randomly selected markers did not show the desired pattern as the ordered marker selection. Therefore, as far as the main effects are concerned, genome selection using the top 27 markers based on LOD scores is the optimal strategy for the soybean embryogenesis trait.

### Epistatic effect model

**Figure 3:**
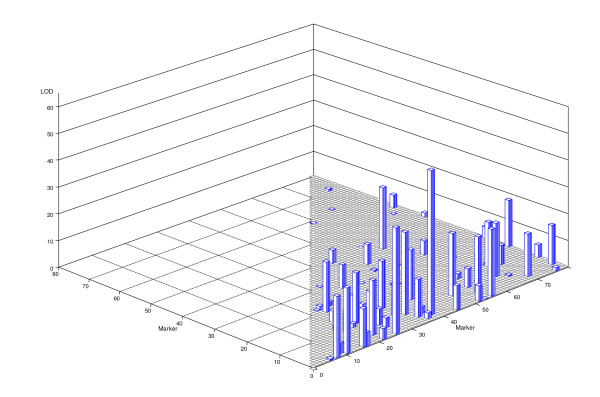
**LOD scores for all the marker pairs (epistatic effects) obtained from the empirical Bayesian analysis**.

The epistatic effect model included 3240 (80 main + 3160 epistatic) effects. The LOD scores of the 3160 epistatic effects are given in Figure 3. The interaction with the largest LOD score happened between markers M3 and M39. This single interaction explained 6.5% of the phenotypic variance. The interaction with the second largest LOD score occurred between M1 and M26, explaining 4.5% of the variance. The top 66 effects and their LOD scores are given in Table 3 (see the cross validation analysis for the epistatic model). Collectively, the top 66 effects explained 77% of the phenotypic variance. When the model included this many effects, the estimated values were all shrunken toward zero to a large degree. All the selected top 66 effects were epistatic effects and the main effects were all shrunken to very small values compared with the epistatic effects. In facts, their effects were absorbed by the epistatic effects due to correlation between the marker genotype indicator variables and the marker pair genotype indicator variables under linkage.

**Table 3:** The empirical Bayesian estimates of the top 66 marker (main) effects and marker pair (epistatic) effects.

Marker 1	Marker 2	Variance	Effect	StdErr	LOD	p- value	H
M3	M39	0.5519	0.7409	0.0476	52.60	0.0000	0.0648
M1	M26	0.4070	0.6365	0.0479	38.29	0.0000	0.0469
M7	M35	0.2960	-0.5417	0.0460	30.06	0.0000	0.0324
M1	M56	0.2342	-0.4832	0.0453	24.66	0.0000	0.0286
M1	M11	0.2163	0.4633	0.0446	23.42	0.0000	0.0263
M37	M59	0.1847	0.4280	0.0414	23.23	0.0000	0.0230
M7	M50	0.2234	-0.4710	0.0456	23.11	0.0000	0.0262
M1	M8	0.1722	-0.4127	0.0412	21.78	0.0000	0.0214
M4	M55	0.2026	-0.4468	0.0454	20.99	0.0000	0.0231
M8	M65	0.1768	0.4174	0.0436	19.87	0.0000	0.0223
M4	M22	0.1574	0.3950	0.0413	19.85	0.0000	0.0194
M11	M24	0.1484	0.3826	0.0414	18.51	0.0000	0.0189
M12	M42	0.1273	-0.3549	0.0385	18.39	0.0000	0.0157
M20	M24	0.1551	-0.3913	0.0426	18.32	0.0000	0.0194
M18	M80	0.1854	-0.4284	0.0475	17.67	0.0000	0.0215
M13	M34	0.1437	0.3772	0.0422	17.34	0.0000	0.0185
M13	M68	0.1175	0.3407	0.0387	16.82	0.0000	0.0156
M3	M70	0.1521	0.3866	0.0451	15.94	0.0000	0.0183
M5	M22	0.1252	0.3515	0.0418	15.37	0.0000	0.0153
M4	M79	0.1377	0.3681	0.0445	14.82	0.0000	0.0167
M4	M36	0.1016	0.3166	0.0395	13.91	0.0000	0.0120
M1	M16	0.1602	0.3975	0.0499	13.74	0.0000	0.0192
M8	M33	0.0941	-0.3046	0.0409	12.04	0.0000	0.0121
M2	M9	0.0920	0.3002	0.0434	10.37	0.0000	0.0105
M12	M24	0.0799	0.2789	0.0413	9.91	0.0000	0.0101
M27	M36	0.0748	0.2701	0.0420	8.97	0.0000	0.0094
M1	M45	0.0741	0.2695	0.0420	8.93	0.0000	0.0090
M33	M50	0.0682	0.2575	0.0435	7.61	0.0000	0.0087
M9	M19	0.0696	-0.2599	0.0443	7.46	0.0000	0.0089
M12	M71	0.0563	0.2338	0.0404	7.28	0.0000	0.0066
M8	M56	0.0630	0.2465	0.0434	7.01	0.0000	0.0077
M11	M26	0.0696	0.2596	0.0483	6.27	0.0000	0.0077
M7	M27	0.0702	-0.2605	0.0499	5.92	0.0000	0.0081
M1	M52	0.0558	-0.2322	0.0451	5.76	0.0000	0.0061
M28	M63	0.0500	0.2187	0.0437	5.43	0.0000	0.0061
M34	M38	0.0506	0.2203	0.0449	5.22	0.0000	0.0059
M53	M79	0.0520	-0.2232	0.0461	5.08	0.0000	0.0059
M39	M45	0.0495	0.2178	0.0451	5.05	0.0000	0.0055
M9	M80	0.0380	-0.1907	0.0407	4.76	0.0000	0.0043
M1	M17	0.0446	-0.2069	0.0442	4.75	0.0000	0.0051
M25	M80	0.0367	-0.1869	0.0411	4.48	0.0000	0.0042
M15	M21	0.0408	-0.1971	0.0449	4.18	0.0000	0.0051
M1	M22	0.0417	-0.1987	0.0471	3.86	0.0000	0.0050
M2	M10	0.0288	0.1643	0.0394	3.78	0.0000	0.0032
M5	M27	0.0330	-0.1769	0.0436	3.57	0.0001	0.0040
M14	M26	0.0347	-0.1810	0.0447	3.55	0.0001	0.0040
M5	M38	0.0232	-0.1454	0.0442	2.35	0.0010	0.0025
M11	M18	0.0217	-0.1408	0.0446	2.16	0.0016	0.0025
M2	M37	0.0240	-0.1480	0.0469	2.16	0.0016	0.0024
M13	M58	0.0193	0.1321	0.0428	2.07	0.0020	0.0023
M25	M53	0.0176	-0.1259	0.0421	1.94	0.0028	0.0019
M44	M80	0.0193	0.1305	0.0474	1.65	0.0059	0.0018
M22	M27	0.0132	-0.1074	0.0404	1.53	0.0079	0.0015
M10	M22	0.0136	0.1086	0.0411	1.51	0.0083	0.0016
M22	M24	0.0098	0.0921	0.0363	1.39	0.0112	0.0011
M1	M77	0.0135	-0.1061	0.0453	1.19	0.0193	0.0013
M71	M76	0.0105	-0.0930	0.0425	1.04	0.0288	0.0010
M29	M48	0.0071	0.0738	0.0415	0.68	0.0759	0.0007
M6	M67	0.0049	-0.0604	0.0364	0.60	0.0976	0.0005
M20	M45	0.0057	0.0639	0.0401	0.55	0.1114	0.0005
M1	M6	0.0037	-0.0509	0.0344	0.48	0.1385	0.0003
M22	M66	0.0035	0.0475	0.0359	0.38	0.1858	0.0003
M19	M47	0.0029	0.0419	0.0337	0.33	0.2141	0.0002
M50	M76	0.0027	0.0396	0.0338	0.30	0.2410	0.0002
M62	M68	0.0021	-0.0338	0.0316	0.25	0.2847	0.0002
M22	M23	0.0027	0.0375	0.0359	0.24	0.2964	0.0002

The last column (H) gives the proportion of phenotypic variance contributed by each effect. The effects are sorted based on their LOD scores.

**Figure 4:**
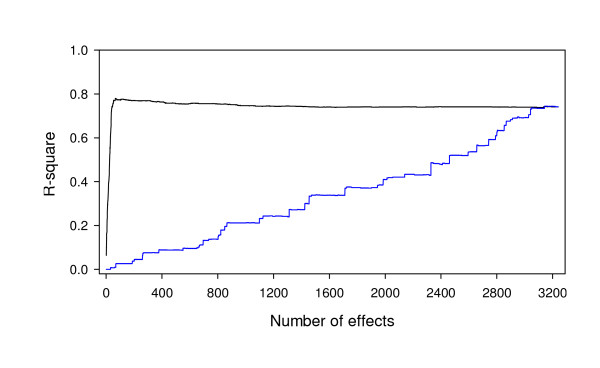
**The r-square between the phenotypic values of somatic embryogenesis and their predicted values**. The black curve represents the change of squared correlation coefficient by increasing the number of sorted (from the strongest to the weakest) effects (markers and marker pairs) in the model. The blue curve shows the change of squared correlation coefficient by increasing the number of randomly selected effects (markers and marker pairs) in the model. The squared correlation coefficient reaches its maximum at 0.78 when 66 effects with the largest LOD scores are included in the model.

The 66 effects were selected from the leave-one-out cross validation analysis. Figure 4 shows the r-square between the observed traits and the predicted genomic values plotted against the number of effects included. When the interaction with the largest LOD score (M3×M39 interaction) was used alone to predict the genomic value, the r-square value was about 0.065. As the number of included effects increased, the correlation increased dramatically and reached the maximum value of 0.78 when the top 66 effects were included. Further increasing the number of effects caused a slight decrease until the correlation reached 0.73 when all effects were included. This explains why the top 66 effects were selected for prediction. Figure 4 also shows the r-square profile (blue) for random inclusion of effects for genome prediction. The curve (in blue) progressively increased until the correlation reached 0.78 that coincides with the black curve for selective inclusion. This pattern is different from that of the main effect model. Further discussion of the r-square profile is provided later in the discussion section.

**Figure 5:**
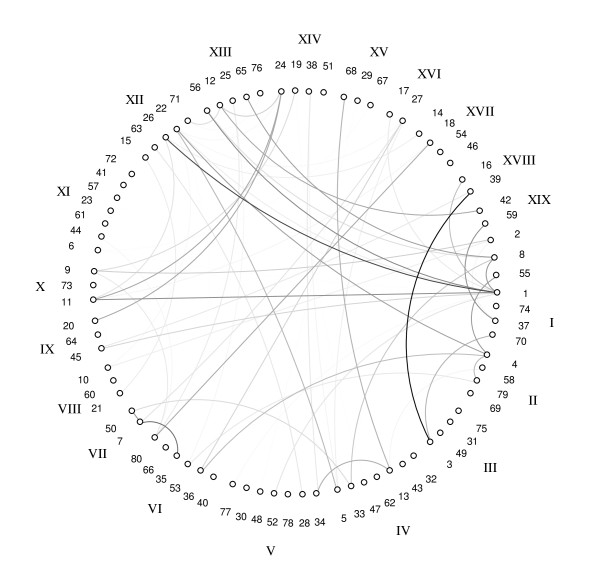
**Epistatic interaction network for the 80 markers investigated in the soybean genome selection mapping project**. The total number of interaction effects is 66. The corresponding names and chromosome positions of the 80 markers can be found in Table 1. The degree of darkness of the lines (epistatic effects) represents the strength of the effects, i.e., darker lines represent stronger epistatic effects.

Figure 5 shows the network of marker interactions where interacting markers are connected with lines whose thicknesses are proportional to the degrees of interactions (LOD scores). Although none of the top 27 main effects showed significant effects when evaluated together with the epistatic effects, 10 of them did appear in the epistatic model as interacting loci.

The conclusion from the epistatic model analysis was that selecting the top 66 effects for prediction, the accuracy (r-square) can reach 78%. If all effects are included, the accuracy decreased slightly to 0.73. Therefore, genome selection using the epistatic model can be very effective in soybean breeding for the embryogenesis trait.

## Discussion and conclusion

We evaluated two models of genome selection for soybean embryogenesis, the main effect model and the epistatic effect model. The main effect model is already very efficient with a 0.33 r-square between the observed phenotypes and the predicted genomic values. With the epistatic effect model, the squared correlation was 0.78, more than twice the efficiency of the main effect model. This discovery provides surprisingly good news to soybean breeders. The squared correlation coefficient between the observed phenotypes and the predicted genomic values is different from the *R*^2 ^value in multiple regression analysis. The r-square here is the squared correlation between the observed phenotypes and the estimated genetic values in which the individual lines predicted contribute to the parameter estimation. The r-square of our epistatic effect model was very high (0.78). In the cross validation generated correlation, the lines predicted do not contribute to the parameter estimation, and thus the squared correlation is a good indication of the predictability. If a new line occurs from the same population but has no phenotypic value, using the marker effects estimated from the existing lines to predict the genetic value of the new line will be as accurate as 0.78. This can save tremendous resources for plant breeding because we can use marker information alone to select plants in several cycles without field evaluation of the phenotypes.

Cross validation analysis showed that the main effect model found 27 important markers, but none of the 27 markers appeared as main effects in the epistatic effect model. This means that epistasis plays a more important role in determining the variation of soybean embryogenesis. These 27 markers did not disappear completely; parts of their effects have been absorbed by the epistatic effects due to correlation of variables in the design matrix. The epistatic model provides better prediction of the genomic values, but it does not disqualify the main effect model. When epistatic effects are excluded from the model, parts of their effects have been absorbed by the main effects due to complicated correlation among the design matrices of the effects. If a breeder decides to use the additive effect model alone for genome selection, he/she still has an accuracy of 0.33.

We used the cross validation analysis to select markers and marker pairs for genome selection. We progressively increased the number of effects in the model to monitor the change in the accuracy of prediction. The optimal number of effects included is the one that maximizes the accuracy of prediction or minimizes the prediction error. The pattern of the change depends on the models used and the traits analyzed. For the main effect model, including more effects than necessary is detrimental to genome selection. For the epistatic effect model, the decrease of the prediction accuracy does not seem to be significant after the optimal number of effects is reached. The reason for the difference in the two models (additive and epistatic) is due to the fact that the large number of effects in the epistatic model caused strong shrinkage of the estimated regression coefficients. Once the prediction accuracy reached the peak value, additional effects included all have extremely small values (virtually equal to zero). Including these zero effects does not cause much damage to the prediction. These patterns of the correlation profiles cannot be generalized to other situations. Therefore, cross validation must be performed for different traits in different experiments.

Unfortunately, the marker density in this experiment was very low in the experiment, which prevented us from using the interval mapping and composite interval mapping approaches for fine mapping. The average interval for the 77 mapped markers was 40 cM. For such a low marker density, we were more concerned about not detecting any QTL. To our surprise, we found more QTL than we originally anticipated. Our cross validation analysis confirmed 27 main effects and 66 epistatic effects. Therefore, we conclude that the soybean embryogenesis is a polygenic trait. The current analysis provides a guideline for further study in fine mapping of QTL for embryogenesis of soybean. The next step is to develop more markers to saturate the entire genome. With a high density marker map, more efficient genome selection can be conducted.

The number of somatic embryos is a discrete trait, which can be modeled by the Poisson distribution. In this particular experiment, each original data point was the average of 10 plants. Therefore, treating the average value as a continuous trait is more reasonable than fitting the Poisson model. For curiosity, we did analyze the data using the Poisson distribution. We also performed a data transformation using the square root of the data [18]. The results were almost identical to the current analysis regarding the markers and marker pairs identified (data not shown).

The main purpose of this study was not to develop new statistical methods for genome selection; rather, we used an existing method [13] to investigate the possibility of using genome selection to improve soybean embryogenesis. The empirical Bayesian method is adequate to handle 80 markers with all pair-wise interactions. When marker density is high, the method may be limited for handling all pair-wise interactions. Past experience [5] showed that the method can handle the number of effects about 60 times as large as the sample size. In this case, the maximum number of effects that can be handled with 126 lines (sample size) is about 126 × 60 = 7560., which is equivalent to about 122 markers. For 1000 markers with roughly 500,000 effects, the sample size should be about 8000 in order to estimate these many effects. When the sample size is limited, improved methods are required. One of the authors in this study (XC) is developing a fast empirical Bayes (fEB) method, which can handle the number of effects as large as 800 times the sample size (unpublished result).

In plant breeding, it is common to select superior cultivars from a random pool of existing accessions. The empirical Bayesian method used in this study works equally well for such randomly selected populations. It is also common to select superior RILs from an RIL crossing experiment derived from two inbred lines [19]. The result of this study can be directly applied to plant breeding using RILs as plant material. In fact, we are in the process of adding more markers and designing a field experiment to apply the selected RILs to improve soybean embryogenesis.

This study demonstrates the importance of epistasis in determination of the embryogenesis of soybean. This finding is clearly in contrast to what Xu and Jia [5] discovered in barley where additive effects play a more important role than epistatic effects in all seven traits investigated in the experiment. However, it is consistent with the study in corn by Dudley and Johnson [9] who discovered that epistatic effects are important in genome selection for yield and oil content traits. Studying the grain yield and its component traits in maize, Ma et al. [20] found that the relative importance of main effects and epistatic effects varies among traits. In addition, they found that only a small proportion of the main-effect QTL interact with other QTL. The study by Yu et. al [21] showed that epistasis also plays a major role in hybrid vigor in rice. This study and the studies by others mentioned above all concluded that main effects and epistatic effects do not have any intrinsic connections. In other words, whether markers interact or not does not depend on the presence of main effects of the two interacting markers. This further supports the notion that epistatic effects and main effects must be studied simultaneously, rather than in sequence where main effects are studied first and epistatic effects are then evaluated only on those markers with significant main effects.

Although there were hundreds of studies in the past decades claiming discovery of major QTL in plants, only a few of them have successfully cloned QTL [22]. QTL mapping and cloning are definitely rewarding. However, many complex traits may be contributed mainly by QTL interactions [9,20,21]. Some may be contributed by polygene only, like the soybean embryogenesis presented in this study. QTL cloning in such cases is obviously not a practical strategy to perform marker aided selection. Fortunately, genomic selection, a special form of marker assisted selection (different from the classical marker assisted selection), has been invented to estimate the molecular breeding values by utilizing saturated markers of the entire genome [2]. Dudley and Johnson [9] further improved the efficiency of genome selection by including epistatic effects in the model. Our study further supports the study of Dudley and Johnson [9]. There is a critical difference between our study and that of Dudley and Johnson. They used a partial least squares (PLS) method to predict the total genomic values of plants, in which marker-trait association was indicated by the association of a few principal components. Our study, however, evaluated the association directly through the association of observed phenotype and marker genotypes, making the method feasible in practical breeding program.

The entire data analysis was conducted using the QTL procedure in SAS, called PROC QTL [23]. This program can perform QTL mapping for not only continuous traits but also discrete traits, such as binary, ordinal, binomial and Poisson traits. In addition, the QTL procedure provides an opportunity for the users to choose maximum likelihood, least squares, weighted least squares or Bayesian method for interval mapping, composite interval mapping or multiple interval mapping. Furthermore, the procedure can analyze multiple traits using the multivariate model. The Bayesian analyses presented in this study were conducted using this QTL procedure. The program is freely downloadable from our website (http://www.statgen.ucr.edu).

## Methods

### Experimental material

The mapping population consisted of 126 F_5:6 _recombinant inbred lines (RILs) that were advanced by single seed descent from the cross of Peking (higher primary and secondary embryogenesis) and Keburi (lower primary and secondary embryogenesis) parents. This population was evaluated for primary embryogenesis capacity from immature embryo cultures by measuring the somatic embryo number per explant. A total of 80 simple sequence repeat markers were available and used for the QTL study. Among the 80 markers, 77 of them were mapped to 19 linkage groups [24]. The marker map was too sparse to perform a meaningful interval mapping. Therefore, we only conducted marker-trait association study, i.e., marker analysis. The remaining three markers were not mapped to any linkage groups, but were subjected to the same marker-trait association study as the 77 linked markers. The phenotype (trait) was measured as the number of somatic embryos per explant. The experiment was replicated in three different plots. Although the number of RILs was 126, the actual number of data points for the three replications was *n *= 3 × 126 = 378. Detailed information about this experiment can be found from Song et al. [24]. To remove the plot effects, each data point was subtracted by the mean of the corresponding plot.

### Empirical Bayesian analysis

#### Main effect model

Genotypes of the Peking cultivar (high embryogenesis) and the Keburi cultivar (low embryogenesis) are denoted by *A*_1_*A*_1 _and *A*_2_*A*_2_, respectively. The genotype of each marker locus was numerically coded and represented by a variable *Z*_*jk *_for the *j*th data point for *j *= 1, ..., *n *and the *k*th marker for *k *= 1, ..., *m*, where *n *= 378 is the number of data points collected from the 126 recombinant inbred lines and *m *= 80 is the number of markers. The numerical coding for *Z*_*jk *_is shown below,

(1) $$Z_{jk}=\{\begin{array}{c} +1\\ -1 \end{array}\begin{array}{c} \text{for}\\ \text{for} \end{array}\begin{array}{c} A_{1}A_{1}\\ A_{2}A_{2} \end{array}$$

The linear model for the plot-mean-adjusted phenotypic value is

(2) $$y_{j}=\beta +\sum_{k=1}^{m}Z_{jk}\gamma_{k}+\varepsilon_{j}$$

where *β *is the population mean, *γ*_*k *_is the effect of the *k*th marker and *ε*_*j *_~ *N*(0,*σ*_2_) is the residual error. The marker effect *γ_k _*is equivalent to the classical definition of the additive effect *a *defined in Falconer and Mackay [25]. Model (2) is called the main effect model, which was used in the Bayesian shrinkage analysis for comparison with the epistatic model.

#### Epistatic effect model

Let *k *and *k*' be two different marker loci, the epistatic effect model is

(3) $$y_{j}=\beta +\sum_{k=1}^{m}Z_{jk}\gamma_{k}+\sum_{k'>k}^{m}Z_{jk}Z_{jk'}\gamma_{kk'}+\varepsilon_{j}$$

where *γ_kk' _*is the epistatic effect between markers *k *and *k*'. The total number of model effects is *m*(*m *+ 1)/2 = 3240, including *m *= 80 main effects and *m*(*m *- 1)/2 = 3160 epistatic effects.

#### Prior distribution

Both the main effect model and the epistatic effect model were analyzed using the empirical Bayesian method [13] implemented in the QTL procedure in SAS [23]. In the empirical Bayesian analysis, each QTL effect, *γ_k _*or *γ_kk'_*, was assigned a normal prior distribution

(4) $$p(\gamma_{k})=N(\gamma_{k}|0,\sigma_{k}^{2})$$

where the variance $\sigma_{k}^{2}$ was further assigned a scaled inverse chi-square prior

(5) $$p(\sigma_{k}^{2})=\text{Inv}-\chi^{2}(\sigma_{k}^{2}|\tau ,\omega )$$

The hyper parameters (*τ*, *ω*) were chosen using the leave-one-out cross validation approach [17]. By trial and error, we found that (*τ*, *ω*) = (-2,0) generated the best result for this data in terms of generating the maximum correlation between the predicted and observed trait values and the minimum prediction error. This set of hyper priors is equivalent to the uniform prior for $\sigma_{k}^{2}$, i.e., *p*($\sigma_{k}^{2}$) = 1.

#### LOD score calculation

We first calculate the Wald-test statistic. For the main effect analysis, the Wald test statistic was

(6) $$W=\frac{{\hat{\gamma }}_{k}^{2}}{\mathrm{var}({\hat{\gamma }}_{k})}$$

For the epistatic effect between loci *k *and *k*', the Wald test statistic was

(7) $$W=\frac{{\hat{\gamma }}_{kk'}^{2}}{\mathrm{var}({\hat{\gamma }}_{kk'})}$$

The *p*-value corresponding to the Wald test was calculated from

(8) $$p-\text{value}=1-F_{\chi^{2}}^{}(1,W)$$

where $F_{\chi^{2}}(1,W)$ is the central *χ*^2 ^distribution with one degree of freedom evaluated at *W*. The Wald test statistic was further converted to the LOD score using

(9) $$\text{LOD}=\frac{W}{2\ln(10)}$$

which was presented in the final report and also used to select markers and marker pairs in the cross validation analyses.

### Cross validation

We used the leave-one-out cross validation approach [17] to evaluating the model and determining the hyper parameter values used in the priors and the optimal number of markers to be included in the model for prediction of the genomic values of the 126 recombinant inbred lines. In the cross validation analysis, we used 125 lines to estimate the parameters and used the estimated parameters from the 125 lines to predict the total genetic effect for the remaining line. Eventually, the genomic value of each line was predicted using the parameters estimated from the other 125 lines. We then calculated the squared correlation coefficient between the observed phenotype *y_j _*and the predicted genomic value ${\hat{y}}_{j}$,

(10) $${\hat{y}}_{j}=\hat{\beta }+\sum_{k=1}^{m^{*}}Z_{jk}^{*}{\hat{\gamma }}_{k}^{*}$$

where γ*_k _is the *k*th effect (either a main effect or an epistatic effect), Z*_jk _is the corresponding design matrix for the *k*th effect and m* is the number of effects included in the model for prediction. The effects were ordered from the highest to the lowest based on their LOD scores. For example, if m* = 15, the model only includes the best 15 effects in the model for prediction. The squared correlation coefficient between the observed and predicted trait values $r_{y\hat{y}}^{2}(m^{*})$ becomes a function of m* for m* = 1, ..., 80 (the main effect model) and for m* for m* = 1, ..., 3240 (the epistatic effect model). The optimal m* is the one that maximizes the squared correlation coefficient $r_{y\hat{y}}^{2}(m^{*})$, where

(11) $$r_{y\hat{y}}^{2}=1-\frac{\sum_{j=1}^{n}{\left(y_{j}-{\hat{y}}_{j}\right)}^{2}}{\sum_{j=1}^{n}{\left(y_{j}-{\hat{y}}_{j}\right)}^{2}+\sum_{j=1}^{n}{\left({\hat{y}}_{j}-\bar{y}\right)}^{2}}$$

is the squared correlation coefficient. Note that this statistic is not the Pearson correlation; rather, it represents the proportion of the phenotypic variance explained by the markers.

## Authors' contributions

ZQ wrote the computer program and performed data analyses. YL, XS and YH provided the experimental material, carried out the molecular genetic studies and participated in the field investigation. XC improved the computer program and helped drafting the manuscript. SX participated in the design of this project, developed the statistical method and drafted the manuscript. WL conceived the project, designed the experiment, supervised students and postdocs involved in the project and revised the manuscript. All authors have read and approved the final manuscript.
